# Differential Androgen Receptor Expression Across Bladder Cancer Stages and Its Link to Poor Outcomes

**DOI:** 10.3390/cancers17243990

**Published:** 2025-12-15

**Authors:** Henning Plage, Nadine Biernath, Kira Furlano, Sarah Weinberger, Jonathan Jeutner, Annika Fendler, Florian Roßner, Simon Schallenberg, Sefer Elezkurtaj, Martina Kluth, Maximilian Lennartz, Andreas Holger Marx, Henrik Samtleben, Margit Fisch, Michael Rink, Marcin Slojewski, Krystian Kaczmarek, Stefan Koch, Nico Adamini, Sarah Minner, Ronald Simon, Guido Sauter, Henrik Zecha, Thorsten Ecke, Thorsten Schlomm, David Horst, Bernhard Ralla

**Affiliations:** 1 Department of Urology, Charité—Universitätsmedizin Berlin, Humboldt-Universität zu Berlin and Berlin Institute of Health, Charitéplatz 1, 10117 Berlin, Germanyjonathan.jeutner@charite.de (J.J.); annika.fendler@charite.de (A.F.); thorsten.schlomm@charite.de (T.S.); 2 Institute of Pathology, Charité—Universitätsmedizin Berlin, Humboldt-Universität zu Berlin and Berlin Institute of Health, Charitéplatz 1, 10117 Berlin, Germany; florian.rossner@charite.de (F.R.); simon.schallenberg@charite.de (S.S.); sefer.elezkurtaj@charite.de (S.E.); david.horst@charite.de (D.H.); 3 Institute of Pathology, University Medical Center Hamburg-Eppendorf, 20246 Hamburg, Germany; m.kluth@uke.de (M.K.); m.lennartz@uke.de (M.L.); s.minner@uke.de (S.M.); r.simon@uke.de (R.S.); g.sauter@uke.de (G.S.); 4 Department of Pathology, Academic Hospital Fuerth, 90766 Fuerth, Germany; andreas.marx@klinikum-fuerth.de (A.H.M.); henrik.samtleben@klinikum-fuerth.de (H.S.); 5 Department of Urology, University Medical Center Hamburg-Eppendorf, 20246 Hamburg, Germany; m.fisch@uke.de; 6 Department of Urology, Marienhospital Hamburg, 22087 Hamburg, Germany; m.rink@marienkrankenhaus.org; 7 Department of Urology and Urological Oncology, Pomeranian Medical University, 70-111 Szczecin, Poland; marcin.slojewski@pum.edu.pl (M.S.); krystian.kaczmarek@pum.edu.pl (K.K.); 8 Department of Pathology, Helios Hospital Bad Saarow, 15526 Bad Saarow, Germany; 9 Department of Urology, Albertinen Hospital, 22457 Hamburg, Germany; nico.adamini@immanuelalbertinen.de; 10 Department of Urology, Helios Hospital Bad Saarow, 15526 Bad Saarow, Germany; thorsten.ecke@helios-gesundheit.de

**Keywords:** bladder cancer, androgen receptor, muscle-invasive urothelial carcinoma, prognostic biomarker, immunohistochemistry, sex differences

## Abstract

**Background**: The androgen receptor (AR) is a hormone-regulated transcription factor known to drive tumor progression in prostate cancer. While AR is also expressed in urothelial carcinoma of the bladder, its prognostic relevance remains unclear due to inconsistent findings and limited cohort sizes. **Methods**: We evaluated AR expression by immunohistochemistry in *n* = 2710 bladder tumors using tissue microarrays, with clinical follow-up data available for *n* = 622 patients. **Results**: AR expression was detected in 22.9% of bladder tumors. In pTa carcinomas, AR positivity decreased significantly from 49.8% in low-grade pTaG2 to 29.1% in high-grade pTaG2 tumors (*p* < 0.0001), with similar levels observed in pTaG3 (31.6%). The lowest AR positivity occurred in muscle-invasive cancers (15.5%, *p* < 0.0001). Within pT2–pT4 tumors, AR positivity was associated with advanced tumor stage (*p* = 0.0014), nodal metastasis (*p* = 0.0060), and AR positivity was significantly linked to reduced overall survival (*p* = 0.0210) with a HR = 1.48 (95%CI 1.06–2.05). In male patients, in particular, the impact was significant (*p* = 0.0042). However, this association was not significant in a multivariate analysis (*p* = 0.188). **Conclusions**: AR expression varied between different stages of urothelial bladder cancers, showing a complex expression pattern. Although AR positivity correlated with adverse outcomes, AR expression alone did not independently predict prognosis.

## Simple Summary

Bladder cancer is more common in men than in women, and sex hormones such as testosterone may influence how the disease develops and progresses. The androgen receptor is a protein that allows cells to respond to these hormones and is well known in prostate cancer, but its role in bladder cancer has been unclear. In this study, we analyzed 2710 bladder cancer samples to find out whether the amount of androgen receptor in tumor cells is linked to patient outcomes. We discovered that men whose tumors had high levels of this receptor lived shorter after surgery than those with little or no receptor expression. These results suggest that the androgen receptor could help identify patients at higher risk and might become a new target for personalized treatment in bladder cancer.

## 1. Introduction

Urinary bladder cancer is the tenth most common malignancy worldwide and represents a major global health burden [1]. Most patients present with non-invasive (pTa) or minimally invasive (pT1) stage cancers, which are characterized by a better prognosis and less harmful treatment strategies, like endoscopic removals or intravesical therapies. Once detrusor muscle invasion occurs, radical cystectomy (RC) remains a standard curative option, yet a substantial proportion of patients still experience early local or distant recurrence despite curative treatment [2]. These patterns highlight the biological heterogeneity of urothelial carcinoma and the need for better markers that reflect tumor aggressiveness and support risk-adapted clinical management.

Bladder cancer also demonstrates one of the strongest sex disparities among solid tumors. Men have a three- to fourfold higher incidence than women, while women tend to present with more advanced disease and experience worse outcomes [3,4,5]. Historically, this discrepancy was attributed mainly to differences in smoking behavior; however, sex ratios have remained stable even as lifestyle habits have converged [6,7]. Thus, sex hormones and their receptors could be potential contributors to the development of bladder cancer [8].

The androgen receptor (AR), a nuclear transcription factor activated by testosterone and dihydrotestosterone, regulates a multitude of androgen-responsive genes [9]. In normal tissues, the highest levels of AR expression occur in the testis and the prostate, but AR expression also occurs in many other tissues [10,11]. AR exerts a broad range of physiological functions in the development and maintenance of the reproductive, musculoskeletal, cardiovascular, immune, neural, and haemopoietic systems [12,13]. In cancer, high-level AR expression commonly occurs in prostate cancer, but many other tumor entities can also express significant levels of AR [14,15]. For bladder cancer, at least 19 studies analyzing between 9 and 472 urothelial carcinomas have described detectable AR expression in 13–77% of cases (Supplementary Table S1). Studies evaluating the clinical role of AR expression in urothelial carcinoma have provided controversial results, however. Two studies in urothelial carcinomas have suggested a link between high AR expression and unfavorable prognosis [16,17], while others could not confirm these findings [18,19,20,21,22,23] or even described associations between AR positivity and favorable disease course [24,25,26,27].

Considering the potential significance of AR expression in urothelial carcinoma, we evaluated the potential prognostic role of AR expression in a previously collected cohort of approximately 2700 urothelial carcinomas in a tissue microarray (TMA) format using a highly validated immunohistochemistry (IHC) assay.

## 2. Materials and Methods

Tissue Microarrays (TMA): The TMAs used in this study were first employed in a recent study on the prognostic role of GATA3 expression in urothelial bladder cancer [28]. Our set of TMAs contained one sample each from 2710 urothelial tumors of the bladder archived at the Institute of Pathology, University Hospital Hamburg; Institute of Pathology, Charité Berlin; Department of Pathology, Academic Hospital Fuerth; or Department of Pathology, Helios Hospital Bad Saarow, and/or treated at Department of Urology, University Hospital Hamburg; Department of Urology, Charité Berlin; Department of Urology, Helios Hospital Bad Saarow; Department of Urology, Albertinen Hospital Hamburg (all in Germany); and Department of Urology and Urological Oncology, Pomeranian Medical University, Szczecin, Poland, between 2005 and 2021. Patients at each center were treated according to the guidelines at the time. In brief, patients with pTa/pT1 disease underwent a transurethral resection of the bladder tumor with or without postoperative instillation therapy, while 1826 patients with pT2–pT4 disease were treated by RC. Available histopathological data, including grade, tumor stage (pT), lymph node status (pN), and status of venous (V) and lymphatic (L) invasion, are shown in Table 1.

**Figure 1:**
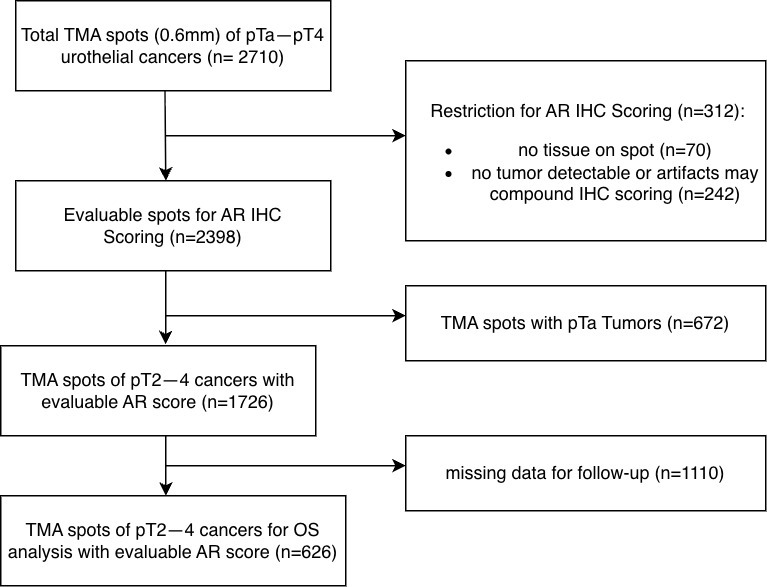
Flow diagram of case selection for survival analysis.

AR immunohistochemistry was evaluable in 2398 of 2710 tumors (88.5%). Non-interpretable cases were due to complete loss of tissue or absence of unequivocal tumor cells on the TMA spot. Among 1826 patients with pT2–pT4 disease who underwent radical cystectomy, overall survival data (time from cystectomy to death or last follow-up) were available for 622 patients (Figure 1). The clinicopathological characteristics of these 622 patients with available overall survival data are summarized in Supplementary Table S4 and were broadly comparable to those of the entire cystectomy cohort (Table 1), indicating that the survival cohort is representative of the underlying pT2–pT4 population. The STROBE-style flow diagram in Figure 1 summarizes case selection, availability of clinicopathological information and survival data, and the numbers of deaths and survivors at last follow-up.

The tissues were fixed in 4% buffered formalin and then embedded in paraffin. The TMA manufacturing process has previously been described in detail [29,30]. Immunohistochemical data on GATA3, p63, CK20, and PD-L1 were available from previous studies [27,29,30,31,32]. Details about the antibody are described in the Supplementary Table S2. All markers were evaluated using the same four-tier IHC scoring system described below. For PD-L1 status, only tumor cells—excluding immune cells—were considered. In brief, one tissue spot (diameter: 0.6 mm) was transmitted from a cancer-containing donor block into an empty recipient paraffin block. The use of archived remnants of diagnostic tissues for TMA manufacturing, their analysis for research purposes, and patient data were according to local laws (HmbKHG, §12); analysis had been approved by the local ethics committee (Ethics commission Hamburg, WF-049/09). All work was carried out in compliance with the Helsinki Declaration.

Immunohistochemistry (IHC).

Freshly prepared TMA sections were immunostained on one day in one experiment. Slides were deparaffinized with xylol, rehydrated through a graded alcohol series, and exposed to heat-induced antigen retrieval for 5 min in an autoclave at 121 °C in pH 7.8 DakoTarget Retrieval Solution™ (Agilent, CA, USA; #S2367). Endogenous peroxidase activity was blocked with Dako Peroxidase Blocking Solution™ (Agilent, CA, USA; #52023) for 10 min. Primary antibody specific against AR protein (rabbit recombinant, MSVA-367R, MS Validated Antibodies, Hamburg, Germany; #2145-367R) was applied at 37 °C for 60 min at a dilution of 1:450. Bound antibody was visualized using the EnVision Kit (Agilent, CA, USA; #K5007) according to the manufacturer’s directions. The sections were counterstained with hemalaun. For each tumor tissue, the percentage of AR-positive tumor cells was estimated, and the staining intensity was semi-quantitatively recorded (0, 1+, 2+, 3+). For statistical analyses, the staining results were categorized into four groups as follows: Negative: no staining at all, weak staining: staining intensity of 1+ in ≤70% or staining intensity of 2+ in ≤30% of tumor cells, moderate staining: staining intensity of 1+ in >70%, staining intensity of 2+ in >30% but in ≤70% or staining intensity of 3+ in ≤30% of tumor cells, strong staining: staining intensity of 2+ in >70% or staining intensity of 3+ in >30% of tumor cells.

Statistics:

Statistical calculations were performed with JMP^®^ 16 software (SAS Institute Inc., Cary, NC, USA). Contingency tables and the chi^2^-test were performed to search for associations between AR immunostaining, other molecular parameters, and tumor phenotype. Survival curves were calculated according to Kaplan–Meier. The log-rank test was applied to detect significant differences between groups. Analysis of variance (ANOVA) was performed to compare the interaction of the parameter sex and androgen receptor expression in the survival outcome of overall survival (OS). Cox proportional hazards regression analysis was used to evaluate the statistical independence and prognostic impact of pathological and molecular variables for both overall survival (OS) and cancer-specific survival (CSS). Pathological tumor stage (pT), pathological nodal stage (pN), venous invasion (V), and IHC androgen receptor status (AR, negative vs. positive) were selected for multivariate analysis (see also Supplementary Table S3). For multivariable CSS models, only covariates that showed a significant association with CSS in univariate testing were entered; therefore, AR status was not included as it did not reach statistical significance in the univariate CSS analysis. In sensitivity analyses, we additionally evaluated alternative dichotomisation schemes (weak vs. negative; low expression [negative + weak] vs. high expression [moderate + strong]) and calculated an immunoreactive score (IRS; range 0–12) based on staining intensity and percentage of positive cells. These analyses are summarized in Supplementary Figure S1.

## 3. Results

**Figure 2:**
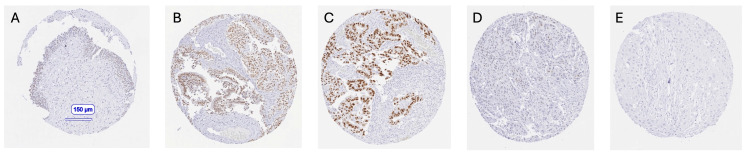
Representative androgen receptor (AR) immunostaining. The panels show moderate nuclear AR immunostaining in normal urothelium (**A**), strong AR staining in a low-grade pTaG2 tumor (**B**), strong AR positivity in a muscle-invasive urothelial carcinoma (**C**), weak positivity in a muscle-invasive tumor (**D**), and complete absence of AR staining (**E**).

Technical issues: Of our 2710 urothelial carcinomas, 2398 (88.5%) were interpretable for AR protein expression analysis. Non-interpretable tumors were caused by a lack of unequivocal tumor cells on the TMA spots or the absence of entire tissue spots on the TMA (Figure 2).

AR in urothelial carcinomas: AR staining was always nuclear. It was usually faint or weak in a subset of normal urothelial cells. Nuclear AR positivity was observed in 22.9% of 2398 urothelial carcinomas, including 399 (16.6%) with weak, 108 (4.5%) with moderate, and 41 (1.7%) with strong staining. AR expression was more common in male (25.9%) than in female (16.2%, *p* < 0.0001) bladder cancer patients. Representative images of AR-positive and AR-negative cancers are shown in Figure 2.

The relationship between AR staining and tumor phenotype is shown in Table 2. Within pTa tumors, the rate of AR positivity decreased markedly from low-grade pTaG2 (49.8% positive) to high-grade pTaG2 (29.1% positive; *p* < 0.0001), while there was no significant further decrease to pTa G3 (31.6%). The AR positivity rate was lowest in muscle-invasive cancers (15.5%; *p* < 0.0001 for all pT2–4 vs. pTa). Among 1726 pT2–pT4 urothelial carcinomas, the distribution of AR immunostaining intensity differed significantly across pT stages (*p* = 0.0014) and according to nodal status (*p* = 0.0060) as well as venous invasion (*p* = 0.0095). There was no statistical relationship with grade or lymphatic invasion.

Survival analysis in pT2–pT4 cystectomy patients

**Figure 3:**
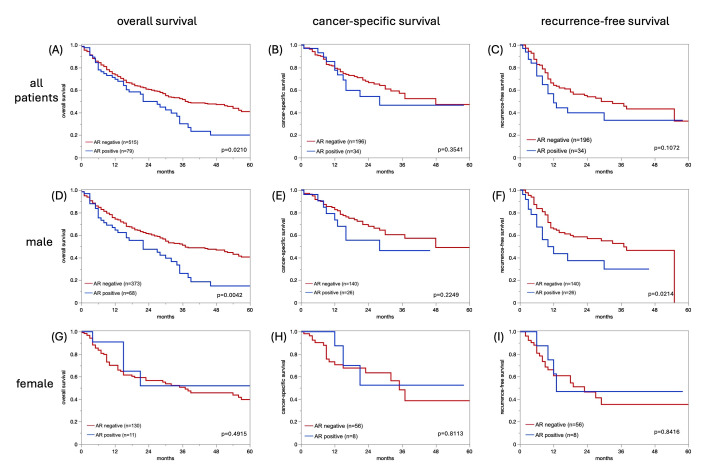
Androgen receptor (AR) immunostaining and patient prognosis with overall, cancer-specific and recurrende-free survival in all patients (**A**–**C**), male (**D**–**F**) and female patients (**G**–**I**).

In the cohort with pT2–pT4 carcinomas that were treated by radical cystectomy, AR positivity was significantly associated with an unfavorable overall survival (OS) in univariate analysis (*p* = 0.0210; HR = 1.48, 95% CI 1.06–2.05; Figure 3A). This effect was mainly driven by male patients, in whom AR-positive tumors had a significantly shorter OS (*p* = 0.0042; Figure 3D), whereas no association was observed in female patients (*p* = 0.4915; Figure 3G). For cancer-specific survival (CSS) and recurrence-free survival (RFS), only AR positivity demonstrated a significant prognostic impact, as shown in the Kaplan–Meier curves (Figure 3B,C,E,F,H,I). A formal interaction test (sex × AR) did not show a significant interaction (*p* = 0.5916).

In the multivariable Cox model for OS, pT-stage, pN-stage, and venous invasion remained independently associated with OS, whereas the association between AR positivity and survival was attenuated and no longer statistically significant (Table 3).

For cancer-specific survival (CSS), Cox models were constructed using the same pathological variables. In line with our predefined strategy, only covariates that showed a significant association with CSS in univariate testing were retained in the multivariable CSS analysis; therefore, AR status was not included as a covariate because it did not reach statistical significance in the univariate CSS analysis. The multivariable CSS models confirm the prognostic relevance of established pathological factors, while not supporting an independent effect of AR on CSS (Table 3).

In additional sensitivity analyses, we explored alternative categorisation schemes for AR expression (Supplementary Figure S1). When survival curves were stratified according to the original four-tier scoring system (negative, weak, moderate, strong), patterns were heterogeneous because only very few tumors showed moderate or strong staining (Supplementary Figure S1A). We therefore evaluated two dichotomisation strategies: weak versus negative expression (Supplementary Figure S1B), and low expression (negative + weak) versus high expression (moderate + strong; Supplementary Figure S1C). These analyses did not materially change the overall association between AR status and outcome. Finally, an immunoreactive score (IRS; 0–12) was derived and dichotomised (IRS 0–1 vs. 2–12), revealing a non-significant trend towards poorer overall survival in IRS-positive tumors (Supplementary Figure S1D).

AR expression and molecular features in bladder cancer: The relationship between AR expression and other molecular features is shown in Table 4. Within pT2–pT4 carcinomas, high AR immunostaining was associated with GATA3 (*p* < 0.0001) and CK20 (*p* < 0.0001) positivity, p63 expression loss (*p* = 0.0013), and low PD-L1 expression in cancer cells (*p* < 0.0001).

## 4. Discussion

The results of this study demonstrate that AR expression occurs in a significant fraction of urothelial neoplasms. AR expression was associated with worse survival in univariate testing, although this effect was not independent of other clinicopathological variables. Nevertheless, the observed trend supports a potential link between AR status and adverse outcomes.

Our findings demonstrate that AR is weakly expressed in normal urothelium and that AR expression is retained and perhaps even enhanced in a fraction of urothelial neoplasms, while most urothelial cancers may either downregulate or completely lose their AR expression. Several earlier studies had also described a progressive loss of AR expression during grade and stage progression of urothelial carcinomas of the urinary bladder [22,23,32,33]. Earlier studies had reported AR positivity rates of 15–70% for low-grade pTa, 22–70% for high-grade pTaG2/3, and 15–52% for pT2–pT4 carcinomas [17,18,21,23,24,25,32,34]. Our AR positivity rate of 49.8% in low-grade pTaG2, 31.6% high-grade pTa, and 15.5% in pT2–pT4 carcinomas is in the lower range of these studies. In contrast to these findings, Elzamy et al. found AR expression to be more frequent in pT2–pT4 (41%) than in non-muscle-invasive urothelial neoplasms (19%) [21]. The largest previous TMA analysis of Mir et al., which included 472 bladder cancer patients, did not demonstrate statistical differences between high and low-grade cancers or non-invasive and muscle-invasive cancers [35]. A significant variability of data is common in IHC analyses if different antibodies, staining protocols, and thresholds for defining positivity are used. Our AR antibody has previously been validated according to the guidelines of the international working group for antibody validation (IWGAV) [36] in 76 different normal tissue categories by comparison with a second independent antibody and with RNA expression data obtained from three different publicly accessible databases (MS submitted).

Another notable finding of our study was the significant association between AR positivity and poor prognosis in muscle-invasive urothelial carcinomas, although this relationship did not remain significant in the multivariate analysis. This observation aligns with several previous reports that did not confirm an independent prognostic role for AR expression [18,19,20,21,22,23]. Nonetheless, a similar trend toward adverse outcomes has been described in other studies. Mashhadi et al. reported a significant association between high AR expression and unfavorable prognosis in a case–control cohort of 120 urothelial carcinomas, although multivariate adjustment was not performed [16]. Toren et al. likewise identified a significant association between elevated AR levels and risk of recurrence in a cohort of 203 muscle-invasive tumors, whereas no relationship with overall survival was observed [17]. Moreover, Sikic et al. found an independent link between high AR mRNA expression and poor prognosis in female patients within a TCGA cohort of 252 muscle-invasive carcinomas, although this analysis was based on transcriptional rather than protein-level data [33]. It should also be noted that these studies differ substantially in methodology, including variations in IHC protocols and scoring systems.

The biological mechanisms underlying the association of AR and aggressive tumor features remain to be elucidated. Epithelial–mesenchymal transition (EMT) may constitute a mechanism by which AR exerts a direct impact on cancer progression. Functional studies have shown reduced tumor growth and EMT measured by the expression of E-cadherin, ß-catenin, and N-Cadherin in AR-suppressed bladder cancer cells in in vitro and in vivo models [37,38]. In addition, one study suggests a role of androgen/AR signaling in phosphorylation of eIF4E, which can promote EMT in bladder cancer cell lines [39].

The prognostic impact of AR expression was more prominent in male than in female patients. This may indirectly argue for a functional role of stimulated AR in urothelial cancer cells with stronger in vivo effects on AR-positive cancers in male individuals with higher serum androgen levels than in females [40,41]. Consistent with our data, Miyamoto et al. had found that carcinogen exposure resulted in higher rates of urothelial cancer development in male than in female mice, while AR knockout mice did not develop bladder cancer, and castration of mice with urothelial carcinoma significantly decreased tumor cell growth [42]. Additionally, AR knockdown in bladder cancer cell lines demonstrated decreased proliferation and migration [43]. Considering the obvious impact of the AR pathway on urothelial cancer cells, it is also tempting to speculate that higher androgen levels in males may contribute to the well-known gender disparity in this cancer type. By contrast, several other studies could not find gender related differences for AR expression in bladder cancer [19,21,22,23,32,35]. In line with the non-significant sex × AR interaction in our formal interaction test, these sex-stratified observations should be regarded as exploratory and hypothesis-generating. Validation in independent cohorts will be required before firm conclusions on sex-specific prognostic effects can be drawn. A clear limitation of our survival analyses is the incomplete follow-up information, as overall survival data were available for only 622 of 1826 patients who underwent radical cystectomy. However, the distribution of stage, grade, nodal status and vascular/lymphatic invasion in the survival cohort was very similar to that of the overall cystectomy cohort (Table 1 and Supplementary Table S4), suggesting that major selection bias is unlikely, although residual bias due to differential loss to follow-up cannot be completely excluded.

The availability of molecular data from earlier studies using the same set of TMAs enabled us to search for possible further effects or interactions of AR expression. The significant association of AR expression with CK20 and GATA3 (markers for luminal bladder cancer) and the inverse correlation with p63, a marker for basal subtype, in muscle-invasive carcinomas is consistent with earlier RNA data linking high AR expression levels to the luminal papillary molecular subtype of urothelial neoplasms, which is overrepresented in men [44]. The mechanism resulting in a striking association between AR expression and a negative PD-L1 status in male patients with muscle-invasive urothelial carcinoma is unclear. However, similar observations have earlier been reported in urothelial carcinoma [45] and in other tumors. In hepatocellular carcinoma (HCC), Jiang et al. [46] demonstrated PD-L1 positivity in >80% of 49 tumors with low AR expression but only in <50% of 25 tumors with high AR expression. In the same study, a direct transcriptional repression of PD-L1 by AR was shown in several HCC cell lines [46]. AR-mediated PD-L1 downregulation was also reported for thyroid cancer [47]. Because the primary aim of our multivariable Cox model was to assess the incremental prognostic value of AR beyond established pathological parameters (pT, pN, venous invasion), PD-L1 was not included as an additional covariate. Consequently, the observed association between AR and PD-L1 should be interpreted descriptively rather than as evidence of an independent prognostic effect, which we acknowledge as a limitation of our analysis.

Based on its common expression in urothelial bladder cancer and its presumed role in cancer progression, therapies targeting AR have been suggested for bladder cancer patients. Interestingly, androgen deprivation therapy (ADT) for prostate cancer or 5-alpha reductase inhibitors for benign prostatic hyperplasia could be linked to a reduced risk of urothelial carcinomas [48]. Further data from several preclinical and clinical studies have also supported the antitumor efficacy of androgen deprivation therapy on AR-expressing urothelial carcinomas [49,50,51,52,53,54,55,56]. In particular, the high expression of AR in non-muscle-invasive cancers might be of therapeutic relevance, as several preclinical studies showed a beneficial effect of the Bacillus Calmette–Guérin (BCG) therapy in the presence of antiandrogens [51,52,54]. A Phase II Trial of bicalutamide in patients receiving intravesical BCG for non-muscle-invasive cancers is currently recruiting (NCT05327647). Despite new agents in bladder cancer, cisplatin-based chemotherapy continues to represent a key systemic treatment option for patients with metastatic or unresectable muscle-invasive urothelial carcinoma. Patients undergoing cisplatin-based chemotherapy may also benefit from antiandrogen therapy, as AR expression has also been linked to cisplatin resistance in urothelial cancers. For example, Kashiwagi et al. found significantly more resistance to cisplatin therapy in urothelial cancer cell lines with higher AR expression than in AR-negative cell lines [53]. In a preclinical study, Tyagi et al. found that enzalutamide contributed as a chemosensitizer, potentiated cisplatin-induced DNA damage in AR-positive bladder cancer cell lines, and still induced apoptosis, and suppressed the migratory and invasive capabilities of bladder cancer cells if the cisplatin dose was reduced [55]. Considering these observations, the combination with enzalutamide could offer a significant advantage to many patients who are ineligible for standard-dose cisplatin due to comorbidities or impaired renal function. In a phase 1b trial (NCT02300610) that evaluated the safety and efficacy of enzalutamide together with cisplatin and gemcitabine in 10 patients with treatment-naïve advanced urothelial carcinoma, 1 patient with a strongly AR-positive tumor showed a complete response, while 4 other patients showed a partial response, and 2 patients had a stable disease [56]. These data support the concept that AR blockade could enhance chemosensitivity to platinum-based regimens in AR-positive bladder cancer. Further research is needed to validate this synergistic interaction and to define the patient subgroups most likely to benefit. Overall, these data underscore the therapeutic relevance of the androgen receptor as a potential target in urothelial carcinoma, particularly in the era of personalized treatment strategies.

## 5. Conclusions

In summary, our data demonstrate significant AR expression in about 20% of urothelial carcinomas and a link between AR positivity and poorer overall survival in male patients with pT2–pT4 disease in univariate analyses, although this association does not remain significant after adjustment for established prognostic factors. Considering also the potential role of AR as a therapeutic target and its potential predictive role for cisplatin resistance, AR is a marker of high interest in bladder cancer that should be further investigated in clinical studies.

## Figures and Tables

**Table 1:** Patient cohort.

	Study Cohort on TMA (*n* = 2710)	
follow up	patients (n)	622
	mean (months)	26.7
	median (months)	15.0
pathological tumor stage	pTa	887 (38.4%)
	pT1	49 (2.1%)
	pT2	462 (20.0%)
	pT3	615 (26.6%)
	pT4	298 (12.9%)
tumor grade	G2	820 (30.6%)
	G3	1858 (69.4%)
lymph node status	pN0	734 (62.0%)
	pN+	449 (38.0%)
resection margin status	R0	595 (80.6%)
	R1	143 (19.4%)
lymphatic vessel infiltration	L0	275 (49.5%)
	L1	281 (50.5%)
blood vessel infiltration	V0	450 (74.4%)
	V1	155 (25.6%)

Percent in the column “study cohort on TMA” refers to the fraction of samples across each category. Numbers do not always add up to 2710 in the different categories because of cases with missing data.

**Table 2:** Androgen receptor (AR) immunostaining and cancer phenotype.

		Androgen Receptor Immunostaining				
	*n*	Negative (%)	Weak (%)	Moderate (%)	Strong (%)	*p*-Value
All cancers	2398	77.1	16.6	4.5	1.7	
pTaG2 low	404	50.2	39.4	9.9	0.5	<0.0001
pTaG2 high	172	70.9	20.9	7.6	0.6	
pTa G3	95	68.4	21.1	5.3	5.3	
pT2	437	82.4	13.3	3.4	0.9	0.0014
pT3	597	90.3	6.4	1.5	1.8	
pT4	289	84.4	10.4	3.1	2.1	
G2	103	88.3	9.7	1.0	1.0	0.6394 *
G3	1194	86.4	9.3	2.6	1.7	
pN0	662	89.9	6.8	2.6	0.8	0.0060 *
pN+	439	84.3	10.3	2.5	3.0	
R0	556	88.7	6.8	2.5	2.0	0.3167 *
R1	140	83.6	10.7	2.1	3.6	
L0	264	90.2	6.1	2.7	1.1	0.2635 *
L1	275	83.6	10.2	2.5	3.6	
V0	429	86.7	7.5	3.5	2.3	0.0095 *
V1	152	87.5	11.2	0.0	1.3	

Abbreviations: pT: pathological tumor stage, G: Grade, pN: pathological lymph node status, R: resection margin status, L: lymphatic invasion, V: venous invasion; * only in pT2–pT4 urothelial carcinoma.

**Table 3:** Multivariate analysis of overall survival (OS) and cancer-specific survival (CSS).

			Multivariate Analysis				
		OS			CSS		
	Covariate	HR	CI (95%)	*p* Value	HR	CI (95%)	*p* Value
	pT-stage	1.58	[1.33–1.89]	<0.0001	1.00	[0.68–1.45]	0.984
both sex	pN-stage	1.57	[1.22–2.01]	<0.0001	1.71	[1.01–2.88]	0.045
	V-status	1.59	[1.02–2.47]	0.039	1.60	[0.79–3.25]	0.191
	AR positivity	1.26	[0.89–1.77]	0.188	n.s. *		
	pT-stage	1.60	[1.32–1.93]	<0.0001	1.14	[0.73–1.78]	0.566
male	pN-stage	1.46	[1.09–1.95]	0. 010	1.53	[0.81–2.91]	0.189
	V-status	1.39	[0.81–2.4]	0.232	6.89	[2.59–18.33]	<0.0001
	AR positivity	1.37	[0.95–1.98]	0.096	n.s. *		
female	pT-stage	1.77	[1.16–2.7]	0.008	2.95	[1.14–7.65]	0.026
	pN-stage	1.96	[1.2–3.2]	0.007	2.82	[1.09–7.28]	0.032
	V-status	1.86	[0.87–3.98]	0.107	n.s. *		
	AR positivity	n.s. *			n.s. *		

Abbreviations: AR: androgen receptor, pT: pathological tumor stage, pN: pathological lymph node status, V: venous invasion, HR: hazard ratio, CI: confidence interval, OS: overall survival, CSS: cancer-specific survival, n.s. * not significant in univariate analysis.

**Table 4:** Androgen receptor (AR) immunostaining and other molecular parameters.

		Androgen Receptor Immunostaining				
	*n*	Negative (%)	Weak (%)	Moderate (%)	Strong (%)	*p*-Value
CK20 negative	736	92.3	5.4	1.6	0.7	<0.0001
CK20 weak	84	85.7	13.1	0.0	1.2	
CK20 moderate	65	70.8	18.5	6.2	4.6	
CK20 strong	375	77.1	16.3	3.7	2.9	
GATA3 negative	481	95.2	3.7	0.2	0.8	<0.0001
GATA3 weak	302	87.1	8.9	3.3	0.7	
GATA3 moderate	237	78.9	16.5	2.5	2.1	
GATA3 strong	171	66.7	21.6	7.0	4.7	
PD-L1 negative	1070	81.1	13.6	3.5	1.9	<0.0001
PD-L1 weak	380	87.9	7.6	2.1	2.4	
PD-L1 moderate	101	94.1	4.0	2.0	0.0	
PD-L1 strong	115	93.9	2.6	1.7	1.7	
p63 negative	200	85.5	8.0	3.0	3.5	0.0013
p63 weak	91	75.8	13.2	8.8	2.2	
p63 moderate	162	90.7	8.0	0.0	1.2	
p63 strong	757	86.5	10.3	2.2	0.9	

## Data Availability

All data supporting the findings of this study are available within the article; additional data are available from the corresponding author upon reasonable request.

## Supplementary Materials

The following supporting information can be downloaded at: https://www.mdpi.com/article/10.3390/cancers17243990/s1, Supplementary Table S1: Summary of previous androgen receptor (AR) immunohistochemistry studies. Supplementary Table S2: Details of the molecular markers used for the comparative analyses. Supplementary Table S3: Univariate analysis of overall survival (OS) in pT2–pT4 urothelial cancers. Supplementary Table S4: Clinicopathological characteristics, including androgen receptor (AR) status, of the overall survival (OS) cohort (n = 622) after radical cystectomy. Supplementary Figure S1: Kaplan–Meier analyses of overall survival (OS) in pT2–pT4 tumors using different AR classification schemes (four-tier score: negative, weak, moderate, strong; dichotomous comparisons: negative vs. weak and low [negative + weak] vs. high [moderate + strong] expression; and immunoreactive score [IRS] 0–1 vs. 2–12).

## Abbreviations

The following abbreviations are used in this manuscript:

ADT	androgen deprivation therapy
AR	androgen receptor
BCG	Bacillus Calmette–Guérin
EMT	Epithelial and mesenchymal transition
HCC	Hepatocellular carcinoma
IHC	Immunohistochemistry
IWGAV	International working group for antibody validation
RC	Radical cystectomy
TMA	Tissue microarray
