# Supplementary material for: Differential Androgen Receptor Expression Across Bladder Cancer Stages and Its Link to Poor Outcomes
Source: Cancers (Basel). 2025 Dec 15;17(24):3990. doi: 10.3390/cancers17243990 (PMC12731148; doi:10.3390/cancers17243990)
Supplement: Supplementary file 1 [file cancers-17-03990-s001.zip › cancers-3979539-supplementary.pdf]

Supplementary Material

Supplementary Table S1. Summary of previous androgen recpetor (AR) immunohistochemistry studies

author/year	PMID	subgroups (histological, therapeutical)	tumors (n)	positive % (all pT and G)	% positive pT≥2	% positive CIS/pT≤1
Birtle et al, 2004	15228457	/	17	52	52	/
Boorjan et al., 2004	15302512	TUR-B/ Cystectomy/ resection metastasis	49	53.1	21.40	75
Kauffman et al, 2011	21400613	Cystectomy only	72	/	/	/
Elzamy et al, 2018	30509050	/	106	34.9	40.5	18.5
Nam et al, 2014	25048477	NMIBC	169	37.2	/	37.2
Mir et al., 2011	21070579	/	472	12.9	15	9
Tuygun et al., 2011	19372057	NMIBC	139	71	21	60
Kim et al., 2015	25755769	NMIBC	118	/	/	/
Izumi et al, 2016	26885620	NMIBC	72	61	/	61
Yonekura et al., 2019	29862474	NMIBC	40	52	/	52
Miyamoto et al., 2012	22221549	/	91	42	/	/
Zhuang et al., 1997	9205860	Cystectomy only	9	77	/	/
Li et al., 2013	23447569	/	24	33.3	/	/
Mumtaz et al., 2020	32104638	NMIBC, Papillary	84	16.7	/	16.7
Tyagi et al., 2019	31006613	/	89	43.8	64 (only male)	44.7
Mashhadi et al., 2014	25433476	/	120	22	/	/
Williams et al., 2015	25218615	/	307	23.7	/	/
Toren et al., 2020	32676741	/	317	49.2	34	59
Shrivastava et al.,2023	37668795	/	132	63.6	50	70.1

Supplementary Table S2. Details to corresponding molecular markers for comparison analysis

Marker	Antibody Type	Clone ID	Dilution
GATA3	mouse monoclonal	MSVA-450M	1:50
CK20	rabbit recombinant monoclonal	MSVA-620R	1:150
p63	recombinant rabbit monoclonal	MSVA-063R	1:150
PDL-1	recombinant rabbit monoclonal	MSVA-711R	1:150

Supplementary Table S3. Univariate Analysis of overall survival OS in pT2-4 urothelial cancers.

Univariate Analysis			
**Covariate**	**HR**	**CI (95 %)**	**p value**
Sex	0.99	[0.75 - 1.3]	0.923
pT-stage	1.73	[1.46 - 2.05]	**<0.0001**
Grade	0.92	[0.59 - 1.43]	0.703
pN-stage	1.85	[1.46 - 2.35]	**<0.0001**
R-status	1.1	[0.61 - 1.97]	0.748
L-status	0.84	[0.56 - 1.27]	0.416
V-status	2.1	[1.36 - 3.23]	**0.001**
AR positivity	1.48	[1.06 - 2.05]	**0.021**

Abbreviations: AR: androgen receptor, pT: pathological tumor stage, pN: pathological lymph node status, R: resection satus V: venous invasion, HR: hazard ratio, CI: confidence interval

Supplementary Table S4. Supplementary Table 4. Clinicopathological characteristics, including androgen receptor (AR) status, of the overall survival (OS) cohort (n = 622) after radical cystectomy

		**AR immunostaining result**				
	n	negative (%)	weak (%)	moderate (%)	strong (%)	p-value
All cancers	622	85.4	9.8	3.1	1.8	
pT2	200	83.0	12.5	4.0	0.5	0.0518
pT3	288	89.2	6.9	1.7	2.1	
pT4	134	80.6	11.9	4.5	3.0	
G2	49	79.6	16.3	2.0	2.0	0.4862
G3	559	86.0	9.1	3.0	1.8	
pN0	340	90.6	6.5	2.6	0.3	**0.0013**
pN+	210	81.0	11.4	3.8	3.8	
R0	184	86.4	7.6	3.3	2.7	0.381
R1	39	82.1	7.7	5.1	5.1	
L0	53	86.8	7.5	5.7	0.0	0.0751
L1	79	78.5	10.1	3.8	7.6	
V0	79	79.7	8.9	6.3	5.1	0.0534
V1	38	86.8	13.2	0.0	0.0	

Abbreviations: pT: pathological tumor stage, G: Grade, pN: pathological lymph node status, R: resection margin status, L: lymphatic invasion, V: venous invasion;

Supplementary Figure S1. Survival analysis (OS) of other AR subgroups in pT2-4 Tumors.

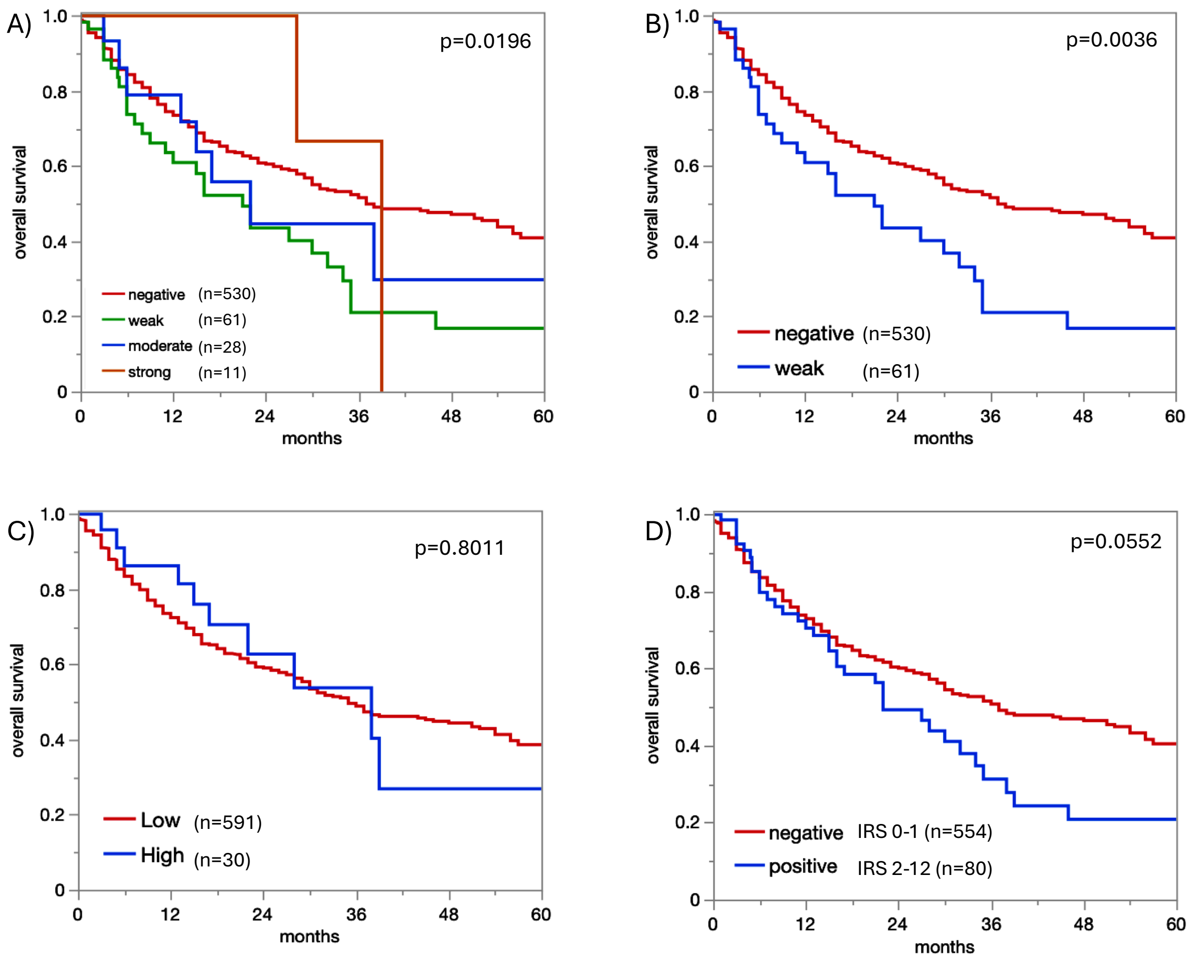

Overall survival was assessed using different AR classification schemes: A) the four-tier system (negative, weak, moderate, strong), B) a dichotomous comparison of only AR-negative versus weak expression, C) an alternative grouping of Low AR (negative + weak) vs. High AR (moderate + strong) AR, D) patient prognosis using the Immunoreactive Score (IRS), calculated with intensity (0–3) × proportion/percentage score (0–4), 0=0%, 1≤10%, 2=10–50%, 3=51–80%, 4≥80% and comparing IRS-negative (0–1) vs. IRS-positive (2–12) tumors. All comparisons were evaluated using the log-rank test.
